# ANCA-Associated Necrotizing Glomerulonephritis Overlapping with Mesangial Proliferative Lupus Nephritis Refractory to Plasmapheresis, Steroid Pulse Therapy, and a Combination of Mycophenolate Mofetil and Rituximab

**DOI:** 10.1155/2018/3076806

**Published:** 2018-11-19

**Authors:** Saika Sharmeen, Clarissa Cassol, Hiroshi Kato

**Affiliations:** ^1^Division of Rheumatology, SUNY Upstate Medical University, Syracuse, NY 13210, USA; ^2^Department of Pathology, SUNY Upstate Medical University, Syracuse, NY 13210, USA

## Abstract

Necrotizing glomerulonephritis (GN) associated with antineutrophil cytoplasmic antibody (ANCA) has been increasingly recognized in the context of class III or IV lupus nephritis (LN), hereafter designated as *ANCA-associated necrotizing LN*. While this subset of GN appears to portend an unfavorable renal outcome, it is not clear whether it represents a distinct entity and benefits from a more aggressive therapy. We report a 78-year-old woman who presented with rapidly progressive GN and was found to have a double-stranded DNA (dsDNA) antibody, hypocomplementemia, antiphospholipid antibody, and strongly positive myeloperoxidase antibody. Renal pathology showed necrotizing and crescentic GN on a background of mesangial proliferative GN. Her kidney disease did not improve despite the treatment with plasmapheresis, three doses of methylprednisolone pulse therapy followed by prednisone at 1 mg/kg/day, rituximab, and mycophenolate mofetil. This case not only reinforces the notion that *ANCA-associated necrotizing LN* is associated with high dsDNA antibody, hypocomplementemia, and worse renal outcome but also adds new insight into the full spectrum of this emerging disease entity by demonstrating that the concurrence of ANCA-associated vasculitis is not specific for class III or IV LN but can also be seen on a background of class II LN.

## 1. Introduction

Glomerulonephritis (GN) is one of the most serious complications of systemic lupus erythematosus (SLE) and ANCA-associated vasculitis as exemplified by so-called *pulmonary-renal* syndrome, yet each disease gives rise to distinct histopathological findings, immune-complex GN, and necrotizing crescentic GN in the absence of significant immune deposits, respectively. Of note, ANCA is detected in approximately 15–20% of patients with SLE [1–4] and in 37% cases of lupus nephritis (LN) [2]. In this respect, necrotizing glomerulonephritis (GN) associated with ANCA has been increasingly recognized in the context of lupus nephritis (LN) [2, 4–7], hereafter designated as *ANCA-associated necrotizing LN*. The vast majority of such cases have been reported in class III or IV LN [2, 4, 6, 7] and were associated with worse renal outcome [2, 4]. However, the entire clinical and histopathological spectrum of this disease entity has not been fully discerned. In particular, it is not clear whether *ANCA-associated necrotizing LN* represents a distinct disease entity or merely overlapping nature of SLE and ANCA-associated vasculitis. Furthermore, treatment approach to this emerging disease category has not been established. Here, we describe a case of an elderly woman who developed rapidly progressive GN with overlapping histopathological features of ANCA-associated necrotizing crescentic GN and mesangial proliferative LN (class II LN) and exhibited refractoriness to aggressive immunosuppressive therapy consisting of plasmapheresis, steroid pulse therapy, rituximab, and mycophenolate mofetil. By reviewing recent findings in immunology research, we discuss potential disease mechanisms of *ANCA-associated necrotizing LN* and their implications for development of target-specific therapeutics.

## 2. Case Presentation

A 78-year-old Caucasian female was admitted to our hospital for a four-month history of severe fatigue, anorexia, and intermittent abdominal discomfort. The patient had been in her usual state of health until four months prior to the presentation when she developed fatigue and abdominal discomfort without apparent inciting factors. She was seen by her primary care physician a week prior to the admission, when she was reportedly found to have severe renal failure; her serum creatinine was 0.74 mg/dL two months prior to the admission. A few days prior to the presentation, the patient started to note dark-colored urine. The abdominal discomfort lacked specific localization and was not postprandial. She had not had hearing loss, vertigo, alopecia, epistaxis, mucosal ulcers, photosensitive rash, pleurisy, dyspnea, hemoptysis, arthralgia, purpura, Raynaud's phenomenon, cutaneous ulcers, muscle weakness, or paresthesia. There was no history of seizures, stroke, coronary artery disease, venous thrombosis, or miscarriages. Her past medical history was only significant for hypertension. Her medications upon admission included metoprolol, losartan, and naproxen. She had no known drug allergies. There was no family history of autoimmune diseases.

On examination, the patient was in moderate distress. The temperature was 97.3°F, the blood pressure was 166/81 mmHg, the pulse was 99 beats per minute, and the oxygen saturation was 98% while she was breathing ambient air. The patient had anasarca. Conjunctivae were pale but not icteric. Her nasal and oral mucosa were normal. There was no tenderness over the sinuses. Superficial lymph nodes were not palpable. Breath sounds were clear in both lungs. There were no pericardial friction rubs. There was no abdominal tenderness or organomegaly. There was no rash. There was no joint tenderness or synovitis. The remainder of the physical as well as neurological examination was unremarkable.

Laboratory studies showed leukocyte count of 4500/*μ*l (reference range: 4000–10,000/*μ*l; reference range is provided in the parentheses in the following laboratory studies), hemoglobin 9.3 g/dL (13.5–18 g/dl), and platelet count 161,000/*μ*l (150,000–400,000/*μ*l). Her leukocyte count, hemoglobin, and platelet count subsequently dropped during her hospitalization and reached their nadirs of 3300/*μ*l, 6.9 g/dL, and 75,000/*μ*l, respectively. Peripheral blood smear did not show schistocytes. Her erythrocyte sedimentation rate was 7 mm/h (0–15 mm/h). Chemistries showed BUN of 54 mg/dL (8–24 mg/dL), creatinine 4.84 mg/dL (0.66–1.25 mg/dL), aspartate aminotransferase 35 U/L (11–39 U/L), alanine aminotransferase 22 U/L (12–78 U/L), alkaline phosphatase 125 U/L (45–117 U/L), albumin 2.5 g/dL (3.2–4.5 g/dL), total bilirubin 0.6 mg/dL (0–1.0 mg/dL), lactate dehydrogenase 224 U/L (84–246 U/L), and haptoglobin 214 mg/dL (30–200 mg/dL). Antinuclear antibody (ANA) indirect immunofluorescence assay was positive at 1 : 250 dilution (homogenous pattern). Solid-phase ANA assay showed positive chromatin antibody and double-stranded DNA (dsDNA) antibody at 71 IU/mL (0–4 IU/mL), while Smith, Ro, La, and RNP antibodies were negative. C3, C4, and CH50 were 46 mg/dL (90–180 mg/dL), 7 mg/dL (10–40 mg/dL), and 116 CAE units (60–144 CAE units). Anticardiolipin IgM was 69 MPL (0–12 MPL) while the remainder of cardiolipin and beta-2 glycoprotein antibodies was negative. Hepatitis B and C serologies did not suggest past exposure. Serum protein and immunofixation electrophoreses did not show paraproteins. Urinalysis showed 3–5 WBC/high-power field (hpf), 50–100 RBC/hpf, and 2+ proteins. A spot urine protein to creatinine ratio was 3.98. The chest X-ray did not show significant parenchymal disease. Based on the ANA, dsDNA antibody, antiphospholipid antibody, cytopenias, and likely GN, the patient was diagnosed with systemic lupus erythematosus (SLE) [8]. In particular, the presentation of her kidney disease, which was consistent with rapidly progressive GN, raised a concern for diffuse proliferative LN, and the patient was started on methylprednisolone 1 gram × 3 doses followed by prednisone 60 mg daily. On the 5th hospital day, ANCA immunofluorescence (IF) assay showed perinuclear staining pattern at 1 : 320 dilution (<1 : 20). Furthermore, myeloperoxidase (MPO) antibody was strongly positive at 147 AU/mL (0–19 AU/mL), while serine proteinase 3 (PR3) IgG was 9 AU/mL (0–19 AU/mL). Accordingly, overlapping ANCA-associated vasculitis was suspected, and she was started on plasmapheresis given its proven benefits in severe renal disease in ANCA-associated vasculitis [9]. However, her BUN and creatinine continued to increase up to 102 mg/dL and 6.08 mg/dL on the 7th hospital day, when she was started on hemodialysis.

**Figure 1:**
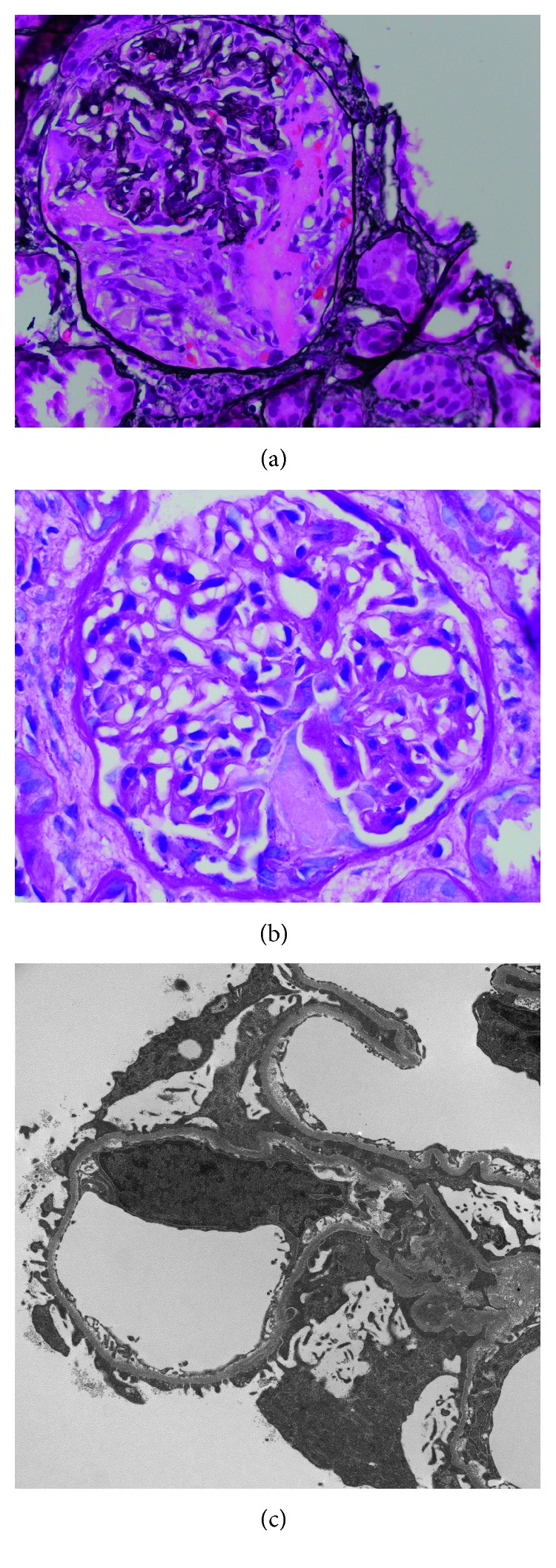
Light microscopy and ultrastructural features. (a) Glomerulus showing a cellular crescent and fibrin within Bowman's space (Jones Silver stain, 400x). (b) Glomerulus with fibrinoid necrosis and segmental mild mesangial hypercellularity (PAS stain, 600x). (c) Electron microscopy showing mesangial electron dense deposits (white arrow).

**Figure 2:**
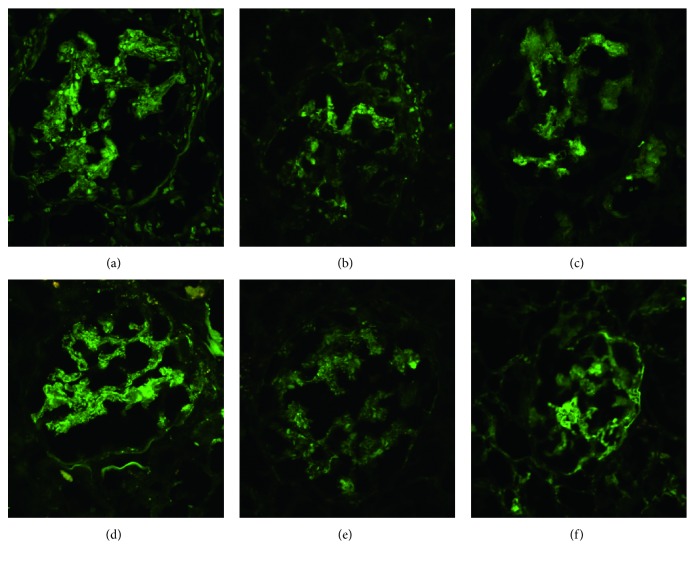
Immunofluorescence microscopy. Granular predominantly mesangial staining for the immunoglobulins (a) IgG (1+), (b) IgM (1+), and (c) IgA (1+) and complement components (d) C3 (2+) and (e) C1q (1+). (f) Segmental fibrinogen staining in an area of fibrinoid necrosis.

Renal biopsy was done on the 4th hospital day and showed an acute necrotizing and crescentic glomerulonephritis, with ten of fourteen glomeruli showing cellular crescents, often in association with areas of fibrinoid necrosis (Figures 1(a) and 1(b)). Only one glomerulus was globally sclerotic, and none showed segmental scars. The glomeruli had mild mesangial and no endocapillary hypercellularity (Figure 1(b)). Interstitial fibrosis was mild (10–15%), and there was no significant vascular disease. The electron microscopy performed on glomeruli extracted from the paraffin block showed predominantly mesangial and rare subendothelial immune-type electron dense deposits (Figure 1(c)). No tubuloreticular inclusions were observed. Immunofluorescence showed mostly mesangial and rare capillary wall weak granular positivity for IgG (1+), IgM (1+), IgA (1+), C1q (1+), and C3 (2+), with equal kappa (1+) and lambda (1+) (Figure 2). There was diffuse nuclear positivity for IgM, IgM, C1q, kappa, and lambda (“tissue antinuclear antibodies” pattern), and fibrinogen highlighted segmental areas of capillary tuft fibrinoid necrosis. The final diagnosis was MPO-ANCA-associated necrotizing and crescentic glomerulonephritis on a background of mesangial proliferative glomerulonephritis consistent with class II lupus nephritis (ISN/RPS).

The patient received a total 6 sessions of plasmapheresis over two weeks. In addition, she received rituximab (375 mg/m^2^ body surface area once weekly) × 3 doses in the hospital and was started on mycophenolate mofetil which was titrated to 1 gram twice daily. We recommended the 4th dose of rituximab as outpatient; however, it was foregone due to difficulty obtaining vascular access. 1.5 months after the initial presentation, the patient was readmitted for severe anemia with hemoglobin of 5.2 g/dL. Her leukocyte and platelet counts were 4,100/*μ*l and 87,000/*μ*l. Her clinical picture did not indicate acute blood loss, and there was no evidence of hemolysis. A chest X-ray did not suggest diffuse alveolar hemorrhage. In association with the foregoing treatment, the titer of her dsDNA Ab declined to 6 IU/ml, whereas her C3 and C4 rose to 80 mg/dL and 14 mg/dL. Her ANCA was undetectable. Collectively, it was felt that SLE was not directly contributing to her anemia, and mycophenolate mofetil was suspended. The patient remained dialysis dependent and subsequently developed sepsis. Given the lack of improvement of overall clinical conditions, the patient elected to pursue comfort care and deceased one month after the second admission.

## 3. Discussion

There is an increasing body of literature reporting necrotizing crescentic GN associated with ANCA on a background of LN, which we designated as *ANCA-associated necrotizing LN* in this article. It is important to note that ANCA in LN is associated with higher SLE disease activity index score [7], worse renal function [6, 7], hypocomplementemia [4–6], dsDNA Ab [2, 6], and worse renal outcome [2, 4], as was exemplified in our case. Histologically, nearly 90% of such cases have been reported in association with class III or IV LN (Table 1) [2, 4–7, 10–18]. In particular, ANCA positivity appears to be associated with higher incidence of class IV-S LN but lower incidence of class V LN, as well as endocapillary hypercellularity [6]. Additionally, ANCA positivity correlates with what is typically observed in ANCA-associated vasculitis including fibrinoid necrosis and crescent formation [5–7]. A series of these observations would allow one to reason that ANCA heralds an aggressive lupus renal pathology. In this regard, it is noteworthy that our case demonstrated mesangial proliferative LN (class II LN), which is virtually never associated with renal insufficiency [19] and has been documented only in 5% of the reported cases (Table 1). While their presenting features and clinical course were not described in most of the previously reported cases of ANCA-associated necrotizing GN overlapping with class II LN, Nasr et al. reported an 80-year-old woman presenting with pulmonary hemorrhage and ANCA-associated crescentic GN overlapping with class II LN who succumbed to her illness despite the treatment with steroid, plasmapheresis, and cyclophosphamide [5]. It is plausible that the component of necrotizing GN, but not that of LN, dictates the renal outcome in such cases.

It is unclear whether *ANCA-associated necrotizing LN* represents a distinct disease entity or merely overlapping nature of ANCA-associated vasculitis and SLE. Although ANCA-associated necrotizing GN has been widely recognized as “pauci-immune” GN, immune-complex GN and ANCA positivity are not mutually exclusive as evidenced by when immune deposits were seen over half of cases of ANCA-associated crescentic GN [20], signifying that the distinction between immune-complex and pauci-immune GN may no longer allow for a precise delineation of glomerulonephritis. In this regard, recent findings in immunology research offer important insight into this potential paradigm shift. Neutrophil extracellular traps (NETs) formation is a cell death pathway characterized by extrusion of chromatin bound to cytosolic and granular contents [21]. Importantly, NETosis has emerged as a salient mechanism that drives lupus nephritis and ANCA-associated vasculitis [22–24]. Low-density granulocytes (LDGs) are a distinct subset of proinflammatory neutrophils found in patients with SLE and exhibit spontaneous NETosis [22]. LDGs externalize immunostimulatory proteins and autoantigens including LL-37, IL-17, and dsDNA and elicit the production of IFN-*α*, one of the signature cytokines in SLE, by plasmacytoid dendritic cells via NETosis [22]. Glomeruli in patients with lupus nephritis are infiltrated by netting neutrophils which expose IL-17 and dsDNA. Along this line, the presence of netting neutrophils in glomeruli was associated with higher titer of dsDNA Ab, class IV as opposed to class III nephritis, and higher activity index on biopsy specimens [22]. Besides the proinflammatory potential of NETosis per se, it is important to note that NETs are not properly cleared from the circulation in patients with SLE which is in part attributed to complement activation [25]. In fact, the inability to degrade NETs is associated with higher incidence of nephritis in patients with SLE [26]. ANCA-stimulated neutrophils release NETs that contain PR3, MPO, and LL-37, and netting neutrophils are infiltrated in kidneys in patients with ANCA-associated vasculitis [24]. It is noteworthy that complement activation and NETosis constitute an self-amplification loop in ANCA-associated vasculitis; i.e., C5a activates neutrophils via C5a receptor, which in turn leads to increased NETosis that activates the complement cascade culminating in production of C5a [23]. A series of these observations implicate increased NETosis and/or defects in NET clearance associated with complement activation as common disease mechanisms shared by lupus nephritis and ANCA-associated vasculitis. Collectively, we reason that *ANCA-associated necrotizing LN* reflects the overlapping disease mechanisms of SLE and ANCA-associated vasculitis rather than a distinct disease entity.

The optimal therapeutic approach for *ANCA-associated necrotizing LN* has not been established. Mycophenolate mofetil has proven to be a comparably effective remission-induction therapy to cyclophosphamide for proliferative LN [27]. Likewise, rituximab has emerged as a promising remission-induction and maintenance therapy for ANCA-associated vasculitis [28, 29]. Furthermore, an observational cohort study suggested a steroid-sparing potential of a combination of rituximab and mycophenolate mofetil in class III, IV, or V LN [30]. Unfortunately, no prospective randomized control trials have been conducted to address the efficacy of aforementioned drugs in *ANCA-associated necrotizing LN*. However, a recent retrospective study demonstrated that mycophenolate mofetil lead to greater frequency of remission and renal survival than cyclophosphamide in ANCA-associated LN [7]. We therefore reasoned that the combination of rituximab and mycophenolate mofetil would be the best available option for this older patient especially in light of the overlapping nature of SLE and ANCA-associated vasculitis. Further studies are clearly warranted to address the efficacy of the foregoing drugs as well as to develop *target-specific* therapy in this aggressive form of GN. As discussed earlier, complement activation plays a role in NETosis which has recently attracted considerable attention as a salient mechanism underlying lupus nephritis and ANCA-associated vasculitis. In this regard, eculizumab, which is a monoclonal antibody against C5 and thereby interferes with C5a/C5a receptor interaction, appeared to be therapeutic in a series of cases of thrombotic microangiopathy (TMA) secondary to SLE [31]. Likewise, C5a receptor inhibitor proved to be steroid-sparing in relapsing ANCA-associated vasculitis in a recent prospective randomized placebo-controlled trial [32].

In summary, we reported a case of ANCA-associated necrotizing GN overlapping with mesangial proliferative LN, but not class III or IV LN, unlike the vast majority of previously reported cases. Her disease did not respond to what we considered was the best available therapy. Increasing body of evidence shed light on NETosis associated with complement activation as a potentially unifying mechanism for SLE and ANCA-associated vasculitis and thereby offers an important hint to fully dissect the clinical and histopathological spectrum of *ANCA-associated necrotizing LN*. We therefore suggest that future studies address the therapeutic potential of neutrophil- and/or complement-targeted drugs in this emerging disease entity.

## Figures and Tables

**Table 1:** ANCA pattern and WHO classification of previously reported cases of ANCA-associated necrotizing LN.

Author	Year	ANCA immunofluorescence assay		ANCA ELISA assay			WHO classification								Total
		*P*	*C*	MPO	PR-3	MPO + PR3	II	III	IV-S	IV-G	V	III + V	IV + V	VI	
Li	2017	N/A	N/A	38	7	4	1	9	10	20	3	2	4	0	
Turner-Stokes	2017	N/A	N/A	26	2	4	2	8	11	7	3			1	
Jarrot	2016	7	0	7	0	0	1			2					
Wang	2016	N/A	N/A	24	2	0	3	6	4		3	5	5	0	
Burkhart	2015	1	0	1	0	0					1				
Abe	2015	N/A	N/A	1	0	0		1							
Isono	2011	N/A	N/A	1	0	0				1					
Morimoto	2010	N/A	N/A	1	0	0				1					
Yu	2009	9	1	7	0	0				7					
Nasr	2008	9	0	5	0	0	1	4	1	1	3	0	0	0	
Hirai	2008	N/A	N/A	1	0	0				1					
Chin	2000	16	3	1	N/A	0		1	17						
Arahata	1999	N/A	N/A	1	0	0			1						
Marshall	1997	1	0	1	0	0					2				
	Total	43	4	115	14	8	8	29	84		15	7	9	1	153^*∗*^

^*∗*^A total number of cases whose WHO classification of LN was available.
